# Seracam: characterisation of a new small field of view hybrid gamma camera for nuclear medicine

**DOI:** 10.1186/s40658-024-00659-7

**Published:** 2024-07-08

**Authors:** Sarah L. Bugby, Andrew L. Farnworth, William R. Brooks, Alan C. Perkins

**Affiliations:** 1 Department of Physics, Loughborough University, Loughborough, UK; 2 Radiological Sciences, School of Medicine, University of Nottingham, Nottingham, UK

**Keywords:** Gamma camera, Portable scintigraphy, Characterisation, Thyroid, Gastric emptying

## Abstract

**Background:**

Portable gamma cameras are being developed for nuclear medicine procedures such as thyroid scintigraphy. This article introduces Seracam® – a new technology that combines small field of view gamma imaging with optical imaging – and reports its performance and suitability for small organ imaging.

**Methods:**

The count rate capability, uniformity, spatial resolution, and sensitivity for ^99m^Tc are reported for four integrated pinhole collimators of nominal sizes of 1 mm, 2 mm, 3 mm and 5 mm. Characterisation methodology is based on NEMA guidelines, with some adjustments necessitated by camera design. Two diagnostic scenarios – thyroid scintigraphy and gastric emptying – are simulated using clinically relevant activities and geometries to investigate application-specific performance. A qualitative assessment of the potential benefits and disadvantages of Seracam is also provided.

**Results:**

Seracam’s performance across the measured characteristics is appropriate for small field of view applications in nuclear medicine. At an imaging distance of 50 mm, corresponding to a field of view of 77.6 mm × 77.6 mm, spatial resolution ranged from 4.6 mm to 26 mm and sensitivity from 3.6 cps/MBq to 52.2 cps/MBq, depending on the collimator chosen. Results from the clinical simulations were particularly promising despite the challenging scenarios investigated. The optimal collimator choice was strongly application dependent, with gastric emptying relying on the higher sensitivity of the 5 mm pinhole whereas thyroid imaging benefitted from the enhanced spatial resolution of the 1 mm pinhole. Signal to noise ratio in images was improved by pixel binning. Seracam has lower measured sensitivity when compared to a traditional large field of view gamma camera, for the simulated applications this is balanced by advantages such as high spatial resolution, portability, ease of use and real time gamma-optical image fusion and display.

**Conclusion:**

The results show that Seracam has appropriate performance for small organ ^99m^Tc imaging. The results also show that the performance of small field of view systems must be considered holistically and in clinically appropriate scenarios.

## Background

Gamma scintigraphy is a well-established diagnostic technique which allows for the clinical and experimental study of physiological processes in the body. Over 100 different diagnostic procedures utilise gamma imaging techniques including processes that demonstrate tissue metabolism (thyroid imaging), organ perfusion (lung and renal studies), vascular drainage (lymphatic imaging), and peptide and antibody binding (tumour imaging) [1].

Gamma cameras used in clinical nuclear medicine departments are typically large devices with a design emphasis on whole body scanning and tomographic imaging. Smaller, portable devices aim to provide flexibility to clinicians and patients, by enabling deployment outside of the nuclear medicine department in configurations that may be more comfortable for patients e.g. close to the patient bedside, or offer enhanced performance for a particular clinical scenario. Mobile systems such as the Ergo (Digirad Health Inc., USA) have a greatly reduced footprint compared to standard gamma cameras, and can be transported around a hospital, but are a far cry from the extremely portable (even handheld) gamma imaging systems that are readily available for applications such as environmental monitoring.

To bridge this gap, a multitude of smaller, portable, pre-clinical devices with a wide range of designs and target applications have been described [2, 3]. Much published research investigates the performance of different elements of these camera systems (e.g. new detector materials, new image analysis techniques) with the development of a portable clinical device as an eventual aim of the work [3]. Several technologies have progressed beyond bench testing to human pilots [2, 4–6], however the vast majority of these systems remain academic or research tools only.

Only four systems have become commercially available to date; the CrystalCam (Crystal Photonics GmbH, Germany) can be purchased and has been demonstrated in several clinical scenarios since 2013 [7–10] though is not cleared for sale as a medical device. The eZ-scope (Anzai Medical Company Ltd., Japan) received 510(k) approval as a medical device in the USA in 2002 but does not appear to be currently commercially available. The IP Guardian 2 (Li-Tech SpA, Italy) is also noted as a commercial device in research articles [11, 12].

The only system currently commercially available and approved as a medical device for clinical use is the Sentinella (Oncovision, Spain). This is sold as a radioguided surgery device and has been demonstrated to be effective extensively in sentinel lymph node biopsy procedures [13, 14] alongside other radioguided surgeries e.g. radioactive seed localisation, radioguided occult lesion localisation (ROLL) [15] and margin assessment [16]. Outside of radioguided surgery, the Sentinella has also been investigated for brain death diagnosis within an Intensive Care Unit [17].

This article introduces a new portable gamma camera system, soon to be commercially available – the Seracam® - and explores its imaging capabilities under standardised characterisation protocols and simulated clinical scenarios.

## Methods

### Seracam imaging system

**Fig. 1:**
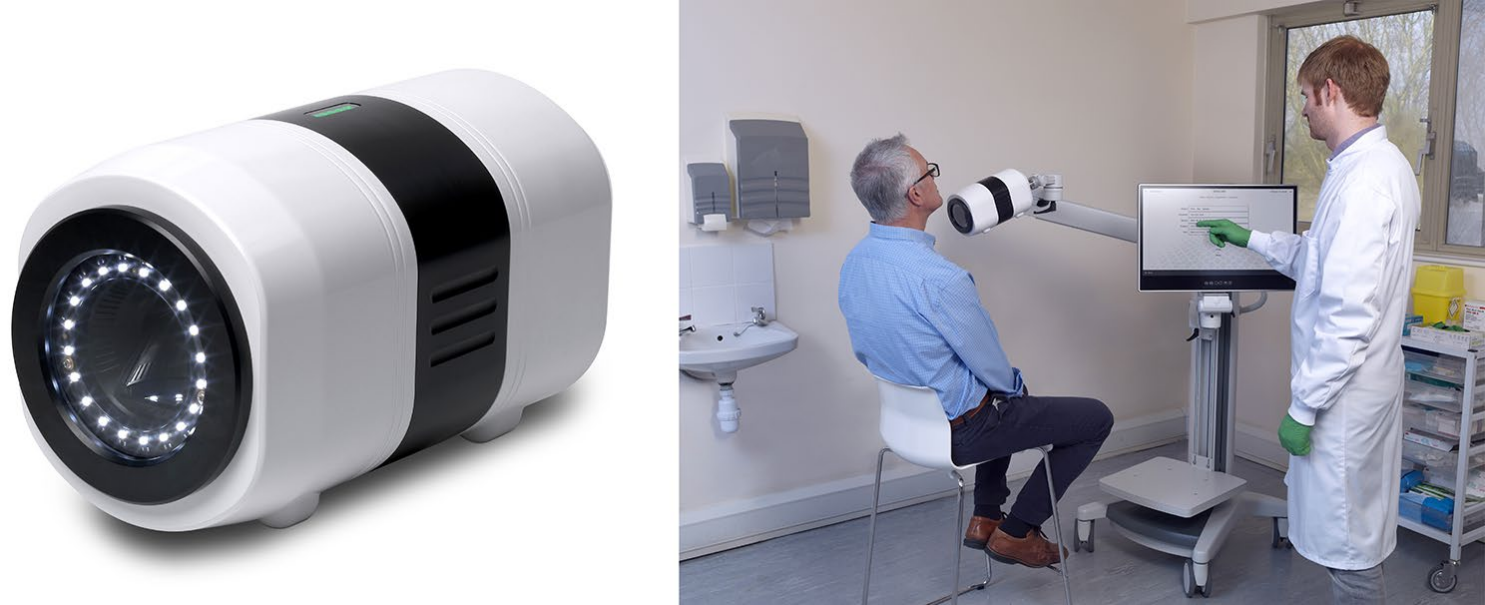
Left: Seracam camera Right: Seracam system. The camera is mounted on a trolley with an articulated arm, data acquisition and display is through the integrated PC with touchscreen display

The Seracam® (Serac Imaging Systems Ltd. UK) is a portable small field of view (SFOV) hybrid optical-gamma camera designed for small organ nuclear medicine imaging procedures (Fig. 1).

The predicate system for the Seracam was based on space research technology [18]and its design [18, 19], performance characteristics [20], and clinically utility [6, 21] have previously been reported. Although Seracam shares some design concepts with this previous device, it has since undergone substantial development and should be considered as a distinct technology.

The Seracam is approximately 15 cm diameter by 24 cm long and weighs 5 kg. It is provided with an articulated arm and trolley for clinical use. A single cable connects Seracam to a power supply and to a computer for data acquisition and display.

#### Gamma detection

Seracam uses a microcolumnar CsI(Tl) crystal scintillator to convert gamma photons to optical photons prior to detection and readout. Each gamma photon absorbed by the scintillator produces a scintillation light splash on a pixellated digital detector.

The imaging area is 25.5 mm square, divided into 245 × 245 pixels of 104 μm pitch. The detector area extends slightly beyond this and its full 256 × 256 pixel area can be read out, however during expected standard operation only the central 245 × 245 pixel imaging area is displayed (see Sect. 2.1.3). For characterisation purposes, we have therefore defined the usable field of view (FOV) as the central 245 × 245 pixels.

Although the readout of scintillation light splashes on the semiconductor is digitised in pixels, the scintillator itself is not pixellated. The light splashes are analysed in real time and the resulting image shows the recorded gamma counts.

Unlike traditional gamma cameras, Seracam uses fixed energy thresholding which is not adjustable by the end user. Seracam’s analysis process has been optimised for ^99m^Tc, and radionuclides with similar energies, and a high pass filter is applied to remove low energy events. The precise analysis method used to generate gamma counts is not publicly available.

#### Collimation

Incoming gamma rays are collimated by a pinhole collimator. The Seracam under test included four different integrated pinhole collimators of nominal diameters 1 mm, 2 mm, 3 mm and 5 mm (actual values measured 1.20 mm, 2.22 mm, 3.07 mm, and 5.00 mm, with a 0.01 mm tolerance), all with an acceptance angle of 60°. Smaller pinhole diameters enhance spatial resolution at the cost of decreased sensitivity [22], and this feature allows easy optimisation of pinhole choice for a given imaging scenario. Where the CrystalCam and IP Guardian 2 use a parallel hole collimator, the choice of a pinhole collimator in Seracam and Sentinella allows for FOVs larger than their imaging detectors.

All four collimators are integrated within the camera head in a rotating carousel. When a collimator is selected within the Seracam software, a motor within the camera head selects and positions the appropriate collimator in front of the detector. This process takes at most a few seconds and does not require the Seracam to be repositioned.

Current nuclear medicine imaging protocols rarely require collimators to be changed within a study - largely due to the practical difficulties of changing collimators quickly and without patient disruption. As the Seracam is not subject to such limitations the choice of collimator can be adjusted to suit the case at hand in real time at the point of care, allowing the preferred balance of spatial resolution and sensitivity to be selected based on the requirements of the imaging.

#### Hybrid gamma-optical imaging

Gamma images in nuclear medicine provide functional rather than necessarily structural information, within the gamma image itself there may be no or very few anatomical landmarks to aid positioning. In standard clinical gamma cameras, the larger FOV can include additional anatomical landmarks (e.g. outline of the body, kidneys) and this is often supplemented with external markers, usually containing a small quantity of ^57^Co or ^99^Tc, placed at anatomical landmarks along the body. The smaller FOV of portable systems, along with their more flexible (and therefore less standardised) positioning option makes anatomical localisation more challenging.

Combined optical and gamma imaging has been proposed as a mechanism to address this [23, 24]. The Sentinella uses a laser crosshair to aid positioning and has more recently added an optical module (originally known as the Horus, but now integrated) [25] – this is offset from the Sentinella pinhole but calibrated to allow optical and gamma registration within a range of standoff distances [26].

**Fig. 2:**
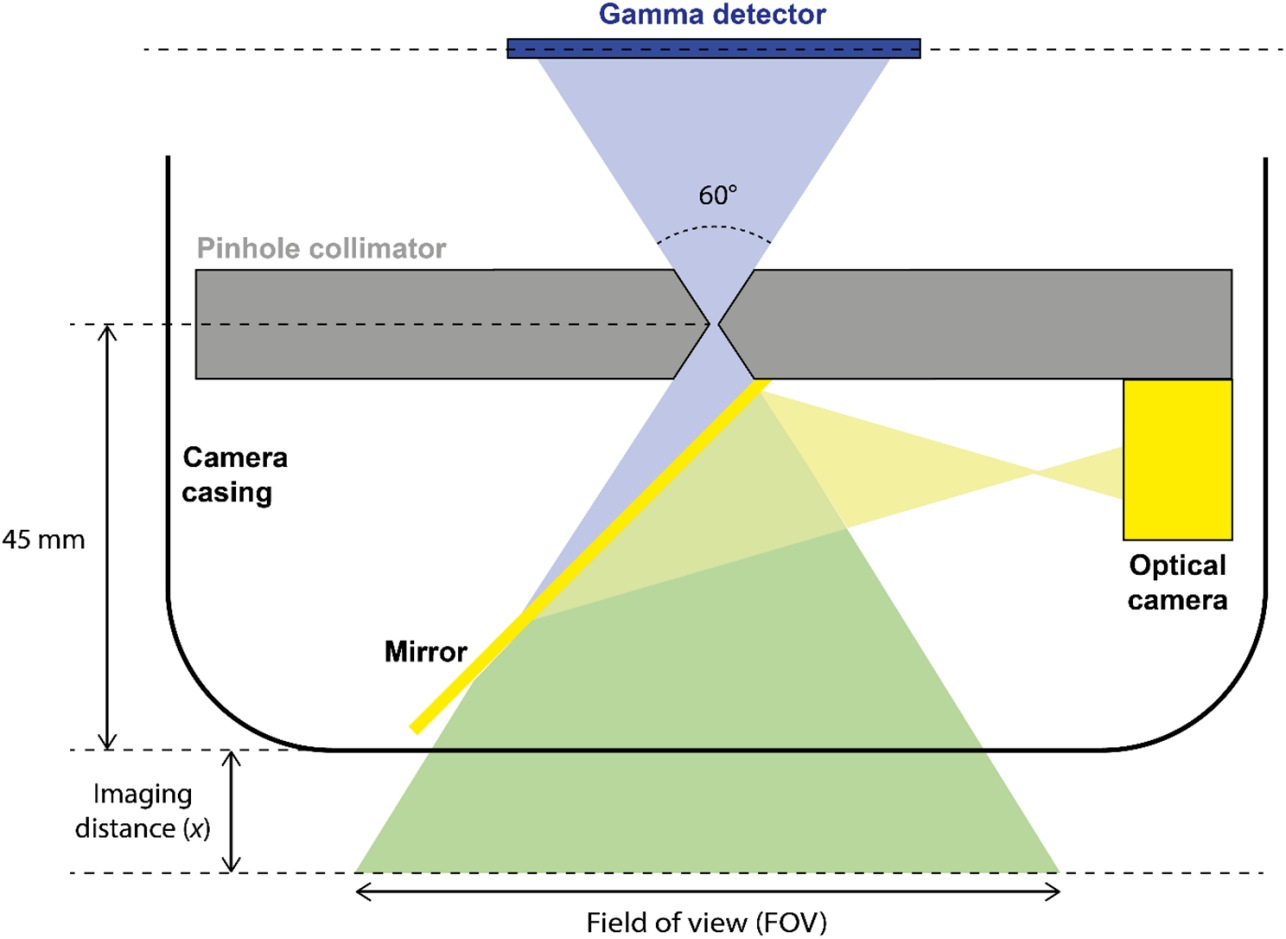
A simplified schematic of the hybrid optical-gamma configuration of the Seracam, showing how co-alignment between optical and gamma images is achieved. Illustration is not to scale

In contrast, the Seracam has a unique co-aligned design for gamma and optical imaging. As illustrated in Fig. 2, a mirror at an angle of 45° is centred over the pinhole and redirects optical light to an optical camera. Gamma photons, however, pass through the mirror with only minor absorption. The position of the gamma detector, pinhole, optical camera, the optical camera’s focal length are chosen to ensure that the magnification ratio between the modalities is constant at any imaging distance, *x.*

As show in Fig. 2, the pinhole collimator is set back 45 mm from the front window of the camera. A pinhole collimator has a circular FOV which is dependent on the imaging distance *x*. In the Seracam, the pinholes have a 60° acceptance angle (i.e. a half angle of 30°) and therefore have a circular FOV with a radius of $\left(x+45\right){tan}\left(30\right)$.

For imaging, a circular display is not used. The displayed FOV is the square inscribed in the circular FOV and therefore has horizontal and vertical dimensions of:

1 $$\text{F}\text{O}\text{V} = \sqrt{2{\left[\left(x+45\right)\text{tan}30\right]}^{2}}$$

This results for example in a FOV of 36.7 mm × 36.7 mm at the camera face (*x =* 0 mm), and 77.6 mm × 77.6 mm at an imaging distance *x =* 50 mm.

#### Readout

The Seracam control software records optical, gamma, and hybrid TIFF images for each acquisition along with the respective DICOM images. An inbuilt database records the time and date of imaging, acquisition length and pinhole choice, along with any user inputted information, for each acquisition. Within the software, the end user may adjust colour tables and contrast to enhance visibility of features, and quantify the number of gamma counts within the image. For research purposes data was recorded as a list of the frame number and pixel location of each recorded photon (i.e. list mode). Recorded image files were exported outside of the Seracam software for detailed analysis, as described in the following sections.

### Characterisation protocols

Characterisation and quality assurance for clinical gamma cameras typically follows protocols developed by the National Electrical Manufacturer’s Association, most recently NEMA NU 1-2018 [27].

These protocols are intended for use with large FOV (LFOV) traditional gamma cameras for which they were designed, but are not always appropriate to the more varied architecture seen in SFOV devices [28] and it is therefore typical for SFOV devices to adjust these protocols [3]. Adjustments are not without precedent; the most recent NEMA guidelines included adaptations for discrete pixel detectors and those with fixed collimators; in the UK, for example, clinical quality assurance typically follows a streamlined version of the NEMA guidelines published by the Institute of Physics and Engineering in Medicine [29]. Throughout the characterisation of Seracam we have endeavoured to follow the spirit of NEMA NU 1-2018 for applicable parameters, even if a precise process could not be replicated. Deviations from NEMA protocols and reasoning for why this is required are explained in the method section for each parameter.

Fillable point source markers (Bright Technologies Ltd., UK) containing 0.1 mL ^99m^Tc-pertechnate were used for point sources. All testing was carried out in a 2 mm thick lead enclosure with dimensions 1.4 m × 0.4 m × 0.8 m – the purpose of this was simply to protect lab users over extended acquisitions. The potential impact of use of the enclosure is enhanced background counts from backscattered photons and marginally fewer external background events e.g. from cosmic rays. For the dimension of the enclosure and activities used, these effects are negligible.

For an end user, the distance from the front of the camera to the gamma source is of most practical interest and so this is the definition of x, imaging distance, used in this report. To enable direct comparisons with other systems the distance from the collimator centre to the gamma source, (a further 45 mm), is also provided.

#### Comparison of Seracam units

Characterisation testing was performed on three Seracam units. There were minor differences in experimental protocol across units (e.g. source activities) and data acquisition software was iterated during the course of testing – neither of which impact the reported results. Each test reported here was confirmed across at least two cameras.

All figures shown in this publication are from a single Seracam unit. The calculated performance characteristics for this unit are shown alongside the experimental uncertainty in this measurement. The range in measurements across all tested devices is then shown in brackets. Data is presented this way, rather than as an average across all units, so that the experimental uncertainties in measurement are not confused with the variation in results across units.

## Performance characterisation

### Count rate capability (CRC)

An ideal imaging system will have a linear relationship between the number of counts incident and the number detected; real systems see this relationship break down at high count rates. The count rate capability (CRC) is the point at which the camera’s response is no longer linear, and cameras should typically only be operated at count rates below this point.

#### Deviation from NEMA protocols

Unlike in traditional gamma cameras, Seracam’s collimators cannot be removed. In this case, NEMA protocols are to use an extended uniformly illuminating (flood) source. Initial testing demonstrated a flood source containing the maximum activity the lab is licensed for was not sufficient to saturate Seracam’s detector. It was possible to reach the fold over limit, for a small area of the detector, when a concentrated point source was used. To enable comparison to other systems, the count rate capability for this small area was then extrapolated across the detector to be a comparable measure to other published work [20, 30, 31].

The number of counts collected deviated from NEMA protocols, as would be expected for a small system.

#### Experimental method

A ^99m^Tc point source (initial activity 357 MBq) was placed at an imaging distance *x =* 150 mm from the Seracam on its central axis. Data was recorded using the 5.00 mm pinhole. The decaying source method [27] was used; list mode data was recorded over a period of approximately 48 h and then split into 15-minute acquisitions for analysis. A 15 min long background acquisition, with no source present, was recorded for background correction.

#### Data analysis

Background and decay correction during each acquisition were performed following NEMA standards [27].

As a pinhole collimator was in place during the acquisition, only a small area of the detector was illuminated. A 25 × 25 pixel square region in the centre of the source image was selected for analysis where illumination was uniform through the pinhole (i.e. far from the pinhole edge). In this region it is assumed that all photons pass directly through the pinhole.

Emitted photopeak count rate (counts per second) was calculated for each acquisition, including the effects of source decay. Incident count rate at the detector was calculated using the source-detector distance, solid angle subtended by the analysed region of the detector, and emitted count rate, to allow for meaningful calculation of source activity limits.

Both incident and detected counts within the analysed region were scaled by a factor of 96.04 to give the expected count rates under complete illumination of the 245 × 245 pixel detector. As defined in NEMA NU 1-2018, we report the maximum recorded count rate and the count rate at which a 20% deviation from a linear response is seen.

#### Results

**Fig. 3:**
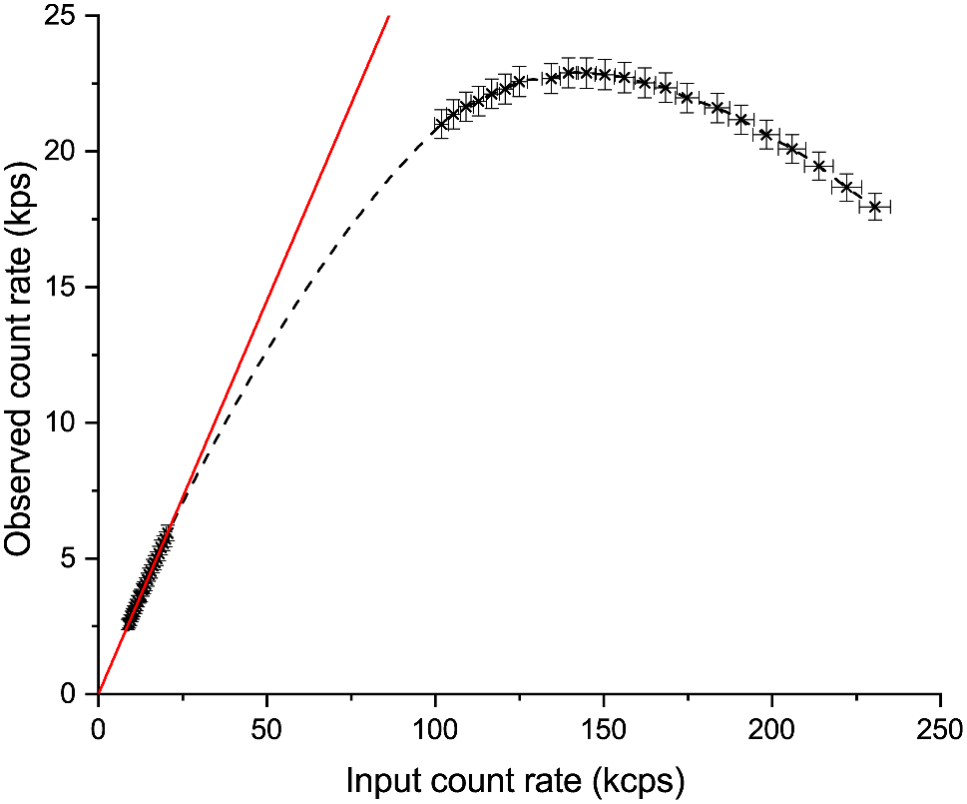
Count rate capability curve for Seracam. Individual data plots shown alongside an interpolated curve (dashed black line). Points below a 50 kcps input rate have been identified as the proportional region for a simple linear fit (solid red line, R^2^ > 0.999). Error bars are derived from uncertainty in initial activity measurement and experimental setup and Poissonian statistics of detected counts

Figure 3 shows a representative CRC curve for Seracam. In the low count region, counts are strongly proportional (R^2^ > 0.9999) and indicate an intrinsic sensitivity of 29%. A 20% deviation from this linear response occurs at an input count rate of 73 ± 1 kcps (ranging from 73 to 78 kcps across units). At high input count rates, Seracam reaches a maximum observed count rate of 22.9 ± 0.1 kcps (peak reached for only one unit).

### Uniformity

An image of a uniform source should itself be uniform, however it is typical for variation in response across the detector to lead to non-uniformity. Uniformity is typically reported as a percentage, with a low uniformity being better (i.e. an image of a uniform source shows less variation). Any calculated uniformity is heavily dependent on the number of counts in an image.

#### Deviation from NEMA protocols

Unlike in traditional gamma cameras, Seracam’s collimators cannot be removed and so the use of a planar source is required per NEMA standards. We use a ^57^Co source rather than ^99m^Tc, this has a similar energy but a longer half-life making it more practical for measurements. NEMA’s suggested pixel size – 6.4 mm – is not appropriate for Seracam. The number of counts collected deviated from NEMA protocols, as would be expected for a small system.

#### Experimental method

Uniformity was measured with a 228.6 mm × 228.6 mm 370 MBq ^57^Co flood source (Eckert and Ziegler Isotope Products, Germany) at *x =* 0 mm using the 5.00 mm pinhole collimator. A deep image was acquired over approximately 12 h (aiming to achieve a mean of at least 1000 counts per pixel). Flat-field correction was applied automatically by the Seracam software.

#### Data analysis

Differential uniformity was calculated following NEMA standards [27], the mean across the image is reported. The coefficient of variation (CoV) - defined as the ratio between standard deviation in counts *σ* and mean counts *µ* – was also calculated.

Measured uniformity is strongly dependent on the photon statistics of the image. Photon counting is governed by Poisson statistics i.e. the expected variance in photon counts, in an ideal system, is equal to the mean number of counts. For a mean number of counts per pixel, we can calculate the Poisson-limited CoV as follows

2 $${CoV}_{Poisson}= \frac{{\sigma }_{Poisson}}{\mu }= \frac{\sqrt{\mu }}{\mu }$$

This is the uniformity that would be measured with an ideal detector, with no instrumental non-uniformity, for the same imaging set. An experimentally measured uniformity would never be expected to be smaller than this.

Pixel binning has a substantial impact on uniformity. Uniformity is calculated for Seracam’s default pixel size (104 μm) and additionally for 5 × 5 pixel binning (equivalent to a 520 μm pixel pitch).

#### Results

Uniformity results across all cameras are summarised in Table 1.

**Table 1:** Uniformity measurements. The results for CAM001 are shown throughout this paper

Parameter	CAM001		CAM002		CAM003	
	No binning	5 × 5 binning	No binning	5 × 5 binning	No binning	5 × 5 binning
Poisson-limited CoV	2.4%	0.6%	6.7%	1.6%	10.1%	2.5%
CoV	4.7%	1.4%	8.3%	2.2%	10.6%	2.8%
Mean differential uniformity	4.7%	1.2%	8.3%	2.1%	10.7%	2.7%

Differences between uniformity measurements across units were dominated by differences in photon statistics. Mean differential uniformity did not differ significantly from the overall CoV. The counts recorded for these measurements are significantly higher than would be expected in a clinical scenario, indicating that any non-uniformity in the Seracam detector is negligible compared to the expected uncertainties due to photon statistics.

When binning to larger pixel sizes, uniformity improved due to enhanced photon statistics.

### System spatial resolution

System spatial resolution describes the ability of the camera to distinguish objects in close proximity to one another. A smaller spatial resolution is better. This will be different for each pinhole and will increase linearly with imaging distance. It will also increase with greater amounts of scattering material or higher photon energies. A better spatial resolution also creates images with better contrast between regions.

#### Deviation from NEMA standards

The NEMA standards specify the use of a line source, but a point source can be used to collect equivalent data [32]. A point source is advantageous for pinhole collimator measurements as there are established analytical models to predict imaging performance. Point source measurements are also common within the small field of view camera development community [3]. The number of counts collected deviated from NEMA protocols, as would be expected for a small system.

#### Experimental method

A ^99m^Tc point source (initial activity 47.6 MBq) was positioned at a fixed distance from Seracam, aligned to its axis, and an image acquired with each pinhole collimator in turn. The position of this source was varied from 100 mm to 430 mm – with only the larger (and therefore more sensitive) pinholes tested at distances > 300 mm. Imaging time varied from 1 min to 10 min, in order to collect at least 1000 counts in each image.

#### Data analysis

For each pixel in the image, the distance from the centre of the feature was calculated. A summed profile (bin width two pixels) was created, plotting distance-from-centre against pixel value – effectively half of the point spread function. This profile was smoothed using a Savitzky-Golay filter of order 2 with a 7 pixel window. Pixel values were normalised to the peak value.

Linear interpolation was used to identify the distance-from-centre at which normalised counts had decreased by 50% and 90% respectively, and these were doubled to find the full-width at half-maximum (FWHM) and full-width at tenth-maximum (FWTM).

Conversion from pixel space FWHM_pixel_ to object space FWHM_obj_ is;

2 $${\text{FWHM}}_{\text{obj}}= \frac{{\text{FWHM}}_{\text{pixel}} \times 104{\upmu }\text{m}}{M}$$

where *M* is the magnification for the given source distance.

#### Results

**Fig. 4:**
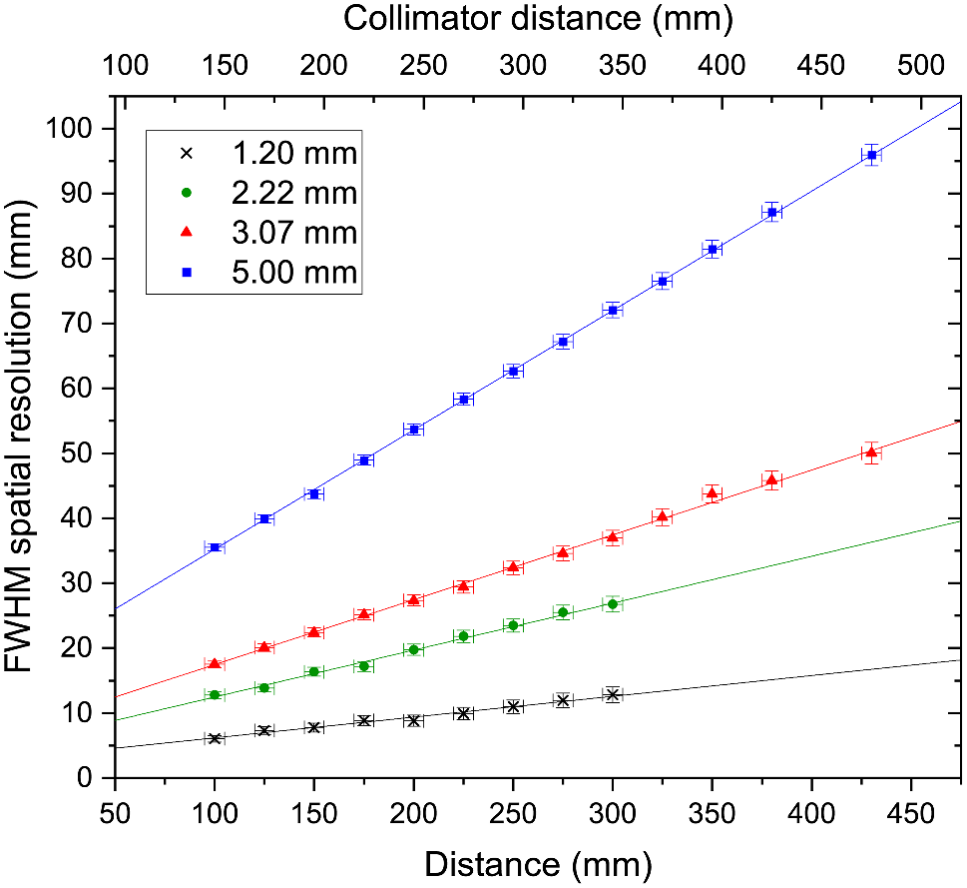
FWHM spatial resolution of Seracam for ^99m^Tc for four different pinhole diameters. Linear fits (R^2^ > 0.98) show the expected relationship between distance and spatial resolution. Error bars derived from uncertainty in source position (including source size) and a 1 pixel error in FWHM_obj_

Figure 4 shows the relationship between FWHM and imaging distance for all four pinholes integrated within Seracam.

The relationship between spatial resolution and imaging distance for on-axis sources follows the form;

3 $$R = {d}_{\text{eff}}\left(\frac{1}{M}+1\right)$$

where *M* is the magnification of the pinhole (the ratio between pinhole-to-detector and pinhole-to-source distances), and *d*_eff_ is the effective diameter of the pinhole, which may be modified to account for the penetration of gamma photons through the collimator material [22, 33].

Fitting this linear relationship produces the following relationships (where *x* is the camera distance).

**Table:** 

1.20 mm pinhole: 2.22 mm pinhole: 3.07 mm pinhole: 5.00 mm pinhole:	[0.032 ± 0.001]*x* + [3.0 ± 0.3] [0.072 ± 0.002]*x* + [5.2 ± 0.4] [0.100 ± 0.001]*x* + [7.5 ± 0.2] [0.183 ± 0.001]*x* + [16.8 ± 0.2]	(4)

with all units tested agreeing within uncertainties. Testing with other units showed this relationship also holds for 0 < *x* < 100 mm.

It should be remembered that *x* is the distance to the camera. Due to Seracam’s unique dual-modality design, the collimator is 45 mm further away from the source than the camera face. If the values in (*4*) are to be used for comparison with other systems this should be considered and, if necessary, *x* modified accordingly. For example, at a 50 mm imaging distance, corresponding to a 77 mm x 77 mm FOV, the spatial resolution of Seracam is 4.6 mm, 8.8 mm, 12.5 mm, and 26.0 mm using the 1.20 mm, 2.22 mm, 3.07 mm, and 5.00 mm pinholes respectively. At a 50 mm *collimator* distance – the measure reported in much of the existing literature and corresponding to a 41 mm x 41 mm FOV – these resolutions are instead 3.2 mm, 5.6 mm, 8.0 mm and 17.8 mm.

### System sensitivity

System sensitivity is the proportion (or percentage) of counts emitted from a point source that will be detected by a camera system. A larger system sensitivity is better as it means smaller amounts of activity can be detected, imaging times can be shorter, or photon statistics can be higher for the same imaging times.

#### Deviation from NEMA standards

The sensitivity measurements here are not equivalent to the NEMA standard for planar sensitivity [27]. Point source measurements, as used here, are common when assessing the performance of small field of view gamma cameras [3].

#### Experimental method

The same data set as for spatial resolution was used (see Sect. 3.3.2).

#### Data analysis

Counts within the image were corrected for background and scaled by imaging time to give a count rate in counts per second. This was then compared to the activity of the source averaged over the acquisition period.

Sensitivity is strongly collimator-dependent and, for pinhole collimators, will decrease with imaging distance with an inverse square law relationship [22]. The expected relationship between imaging distance *x* and sensitivity *S* for a centrally placed source is [22];

5 $$S= \frac{A}{{\left(x+45\right)}^{2}}$$

where *A* is a fitted constant which differs for different collimators [22, 34].

#### Results

**Fig. 5:**
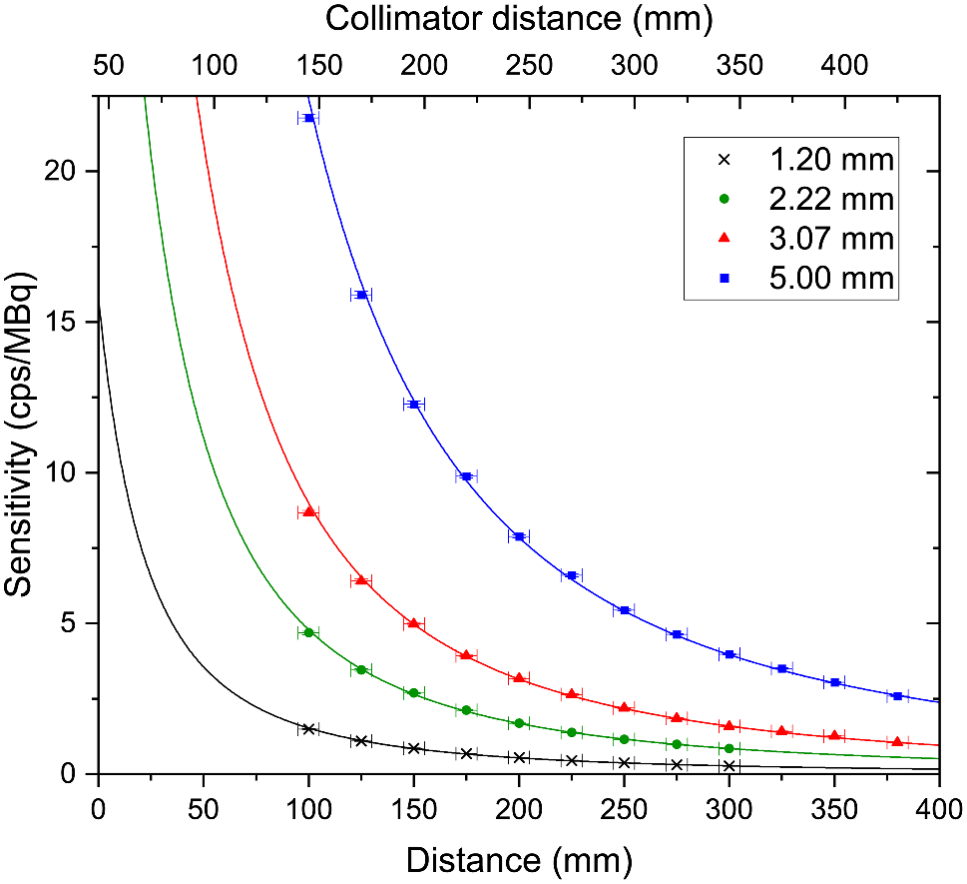
Sensitivity of Seracam for ^99m^Tc for four different pinhole diameters. Fitted curves (R^2^ > 0.998) show the expected inverse square law relationship (Eq. 4). Error bars derived from uncertainty in source position (including source size), initial activity measurement and Poissonian statistics of detected counts

Figure 5 shows the relationship between sensitivity and imaging distance for all four pinholes integrated within Seracam.

Equation 5 was fitted to the data in Fig. 5, the fitted *A* parameters are presented in Table 2.

**Table 2:** Fitted constants from (4) to the Seracam data in Fig. 5

Pinhole	A
1.20 mm	32,100 ± 200
2.22 mm	100,700 ± 500
3.07 mm	189,000 ± 2000
5.00 mm	471,000 ± 3000

As with spatial resolution, the distinction between camera distance and collimator distance is important. At a 50 mm camera distance the sensitivity to ^99m^Tc of Seracam is approximately 3.6 cps/MBq, 11.2 cps/MBq, 20.9 cps/MBq, and 52.2 cps/MBq using the 1.20 mm, 2.22 mm, 3.07 mm, and 5.00 mm pinholes respectively. At a 50 mm *collimator* distance the sensitivities are instead 12.8 cps/MBq, 40.2 cps/MBq, 75.6 cps/MBq and 188.4 cps/MBq.

### Summary of performance characteristics

Table 3 provides an overview of Seracam’s performance characteristics at an imaging distance of 100 mm. As with all pinhole collimators, actual performance will vary significantly with imaging distance. Generally, better performance is achieved for smaller distances between the camera and area of interest.

**Table 3:** Summary of performance characteristics of Seracam for ^99m^Tc at an imaging distance of 100 mm

Parameter	Collimator	Measurement	Result
Field of view	N/A	N/A	118.4 mm × 118.4 mm
Uniformity	N/A	Coefficient of variation (^57^Co)	< 5%
Count rate capability	N/A	Maximum measured count rate	22.9 kcps
		20% deviation from expectation	73 kcps
Spatial resolution	1.20 mm	FWHM	7.6 mm
	2.22 mm		15.6 mm
	3.07 mm		22.0 mm
	5.00 mm		43.3 mm
Sensitivity	1.20 mm	Point source	1.53 cps/MBq
	2.22 mm		4.79 cps/MBq
	3.07 mm		8.99 cps/MBq
	5.00 mm		22.40 cps/MBq

## Experimental simulations of clinical scenarios

### Gastric emptying

A gastric emptying study is used to measure the time it takes for the stomach to empty after ingestion of a radiolabelled meal. Both delayed and rapid gastric emptying can be indicative of a range of conditions requiring treatment [35].

During a gastric emptying study, a patient will ingest a small amount of radioactive material, typically radiolabelled with a ^99m^Tc non-absorbable marker. After ingestion, a series of short (approx. 60 s) images are acquired at regular intervals over the subsequent 1–2 h [35].

A gastric retention curve is then plotted showing the counts at each time point expressed as a percentage of initial intake after correction for background activity and radioactive decay.

#### Method

**Fig. 6:**
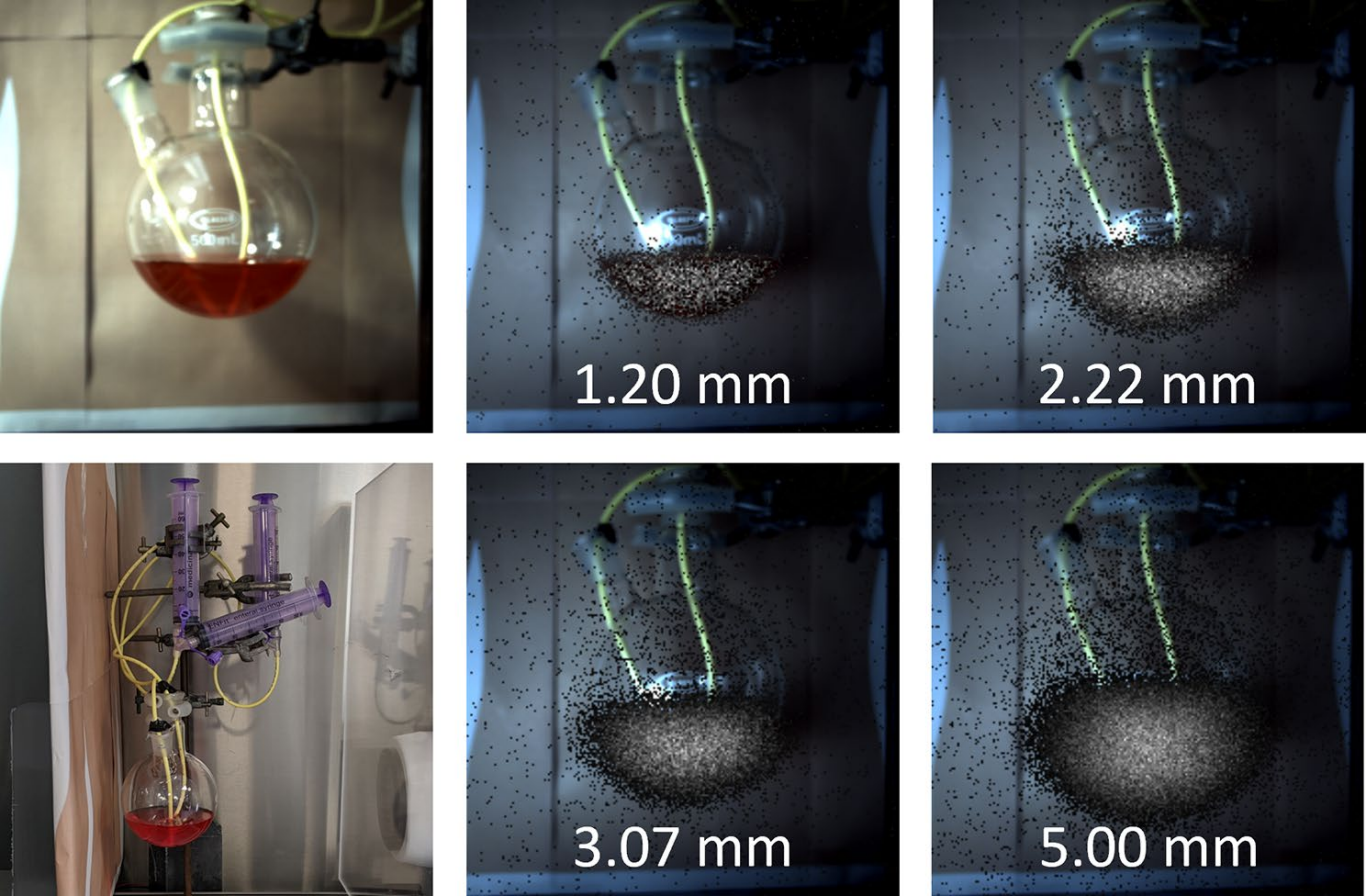
Left: Photographs of the phantom set up showing the round bottom flask, tubing, and syringes. Right: Hybrid Seracam images. In each case the set up was identical, with only the pinhole changed. Images were acquired for 5 min with a total ^99m^Tc activity of approximately 50 MBq. The number of counts within each image is approximately 5300, 15,800, 29,000, 70,000 for the 1.20 mm, 2.22 mm, 3.07 mm and 5.00 mm pinhole respectively. Gamma images are displayed with a default grayscale colour table fused with the Seracam optical image

The human stomach was simulated with a 500 mL round bottom flask. Two nasogastric tubes were inserted into the flask, connected to three 60 mL syringes. Seracam was positioned 120 mm from the front of the phantom, corresponding to a 135 mm × 135 mm FOV, with 60 mm scattering material (Perspex) placed behind the phantom and 15 mm in front to simulate body bulk. This set up is shown in Fig. 6 along with illustrative hybrid Seracam images.

A total of 3.7 MBq ^99m^Tc was diluted into 140 mL. The Nottingham Test Meal standard [35] is 10 MBq in 400 mL, so this choice is in line with clinical practice. Initially, the flask contained the full 140 mL, to simulate the emptying process this was drawn up into the syringes in 6mL increments. A 120 s image was acquired with the Seracam at each step using the 5.00 mm pinhole. The 5.00 mm pinhole was chosen to maximise counts within the short acquisition time.

A region of interest (ROI) was drawn to fully encompass the active area of the phantom and counts within the ROI were recorded for each image. It should be noted that this would be more challenging in a clinical scenario where the patient has moved and been repositioned between images, but this could be overcome with anatomical landmarking.

#### Results

**Fig. 7:**
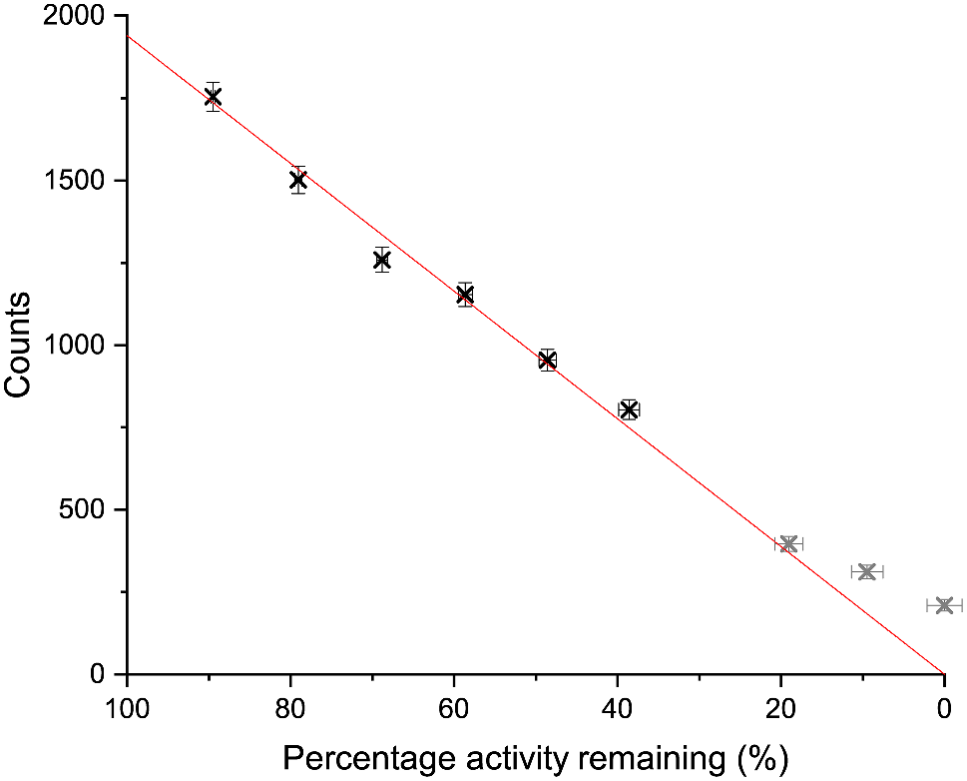
Results from gastric emptying simulation. Initial activity was 3.7 MBq ^99m^Tc in 140 mL, percentage activity remaining was calculated from volume drawn out of phantom and corrected for decay. Error bars derived from uncertainty in volume measurements and Poissonian statistics of detected counts. The linear fit (red line, R^2^ > 0.99) is fitting to the first 7 data points – shown in black - only, with a fixed intercept at 0. Grey data are excluded due as experimental artefacts

Figure 7 shows the simulated gastric emptying curve. The expected linear relationship is clear in the initial data points (to 20% activity remaining). At lower fill percentages deviation from the linear relationship is seen. This is believed to be an experimental artefact as, when only a small amount of liquid remained in the phantom, air was drawn up alongside liquid resulting in an underestimation of remaining activity.

### Thyroid

Thyroid imaging is a long standing diagnostic nuclear medicine investigation for the assessment of the function of thyroid tissue, thyroid nodules and tumours [36].

Alongside radioactive iodine ^131^I and ^123^I - for therapy and diagnosis respectfully - a common tracer used in thyroid imaging is ^99m^Tc-pertechnetate ([^99m^Tc]TcO_4_^−^), which acts as a pharmacologic mimic to iodine and is taken up by follicular thyroid cells. In a typical thyroid study, ^99m^TcO_4_^−^ is administered intravenously and, following a 15–20 min uptake period, the thyroid is imaged from the anterior and lateral directions [36].

The size, location, tracer uptake and uptake pattern within both the thyroid and any small thyroid nodules provide the diagnostic content of a thyroid image. Devices for thyroid imaging must achieve good sensitivity for uptake measurements and excellent spatial resolution to resolve uptake patterns and nodules with abnormal function.

#### Method

##### Picker thyroid phantom

The Picker phantom (Picker International Cleveland, USA) is a common phantom within nuclear medicine, consisting of a 122 mm × 112 mm × 23 mm acrylic block with a 60 mm × 60 mm × 18 mm thyroid-shaped well. The right lobe of the thyroid well is filled with a 9 mm thick plate, reducing the well volume by 50%. The left (18 mm deep) lobe contains 6 mm and 12 mm diameter infills, and the right lobe contains a 9 mm diameter infill and a full depth 12 mm diameter well. The Picker phantom was chosen to provide a reference object familiar within the nuclear medicine community.

The Picker phantom was filled with 17.8 MBq of ^99m^Tc. Images were acquired from an imaging distance of 50 mm, so that the entire phantom was within the FOV, using the 1.20 mm diameter pinhole collimator.

##### Loughborough Head and Neck phantom (LHNP)

**Fig. 8:**
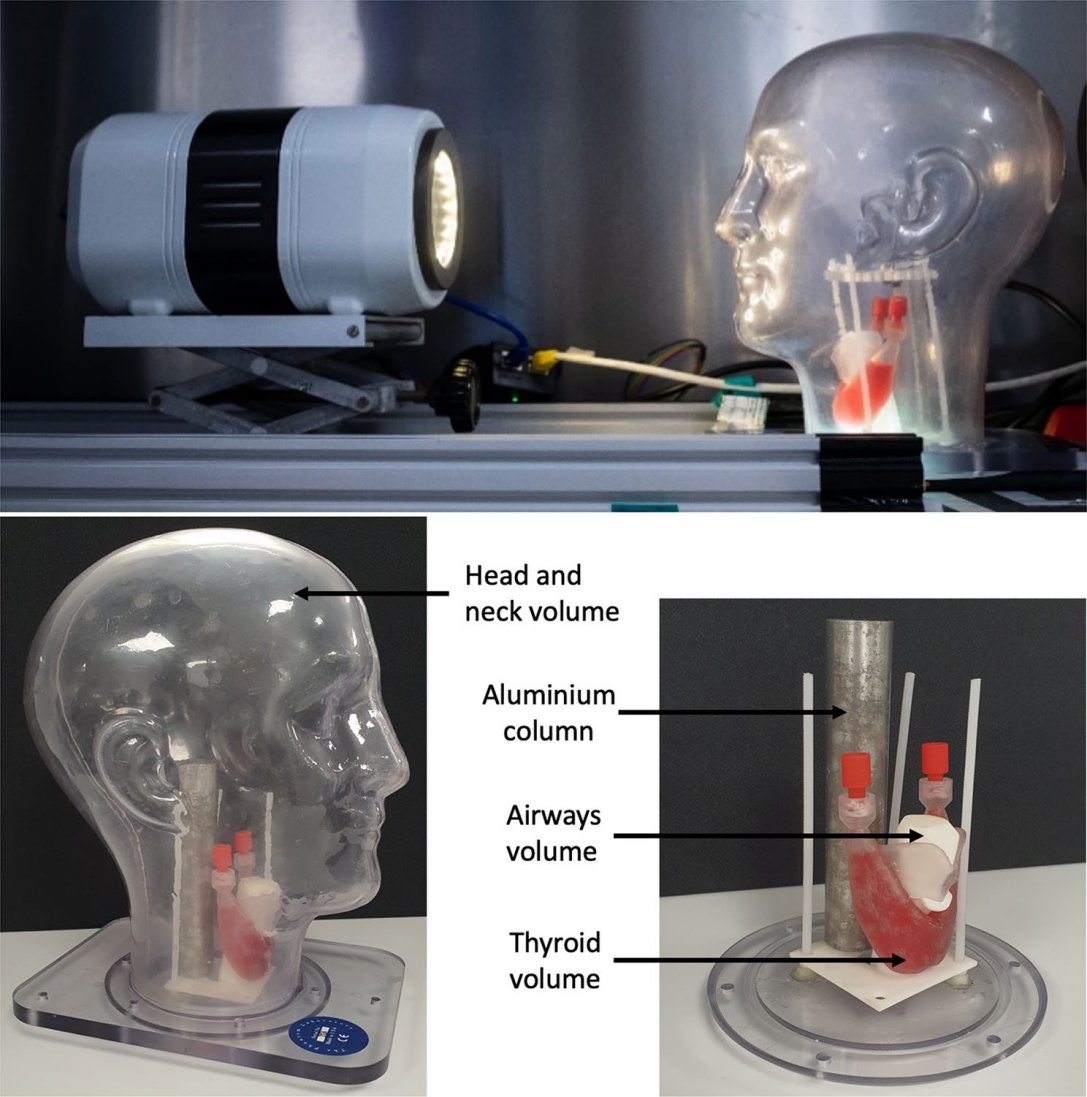
Images of the Loughborough head and neck phantom. During data acquisition, Seracam was centred on the thyroid volume at a distance of 50–65 mm from the phantom

The LHNP was developed to produce clinical-like thyroid images to allow qualitative image quality assessment. This phantom consisted of an anthropomorphic fillable head volume (Phantom Laboratory, Salem, USA) with internally mounted fillable thyroid and hollow airway volumes. The dimensions of the fillable thyroid were 50 mm × 60 mm × 25 mm, resulting in an approximately 13 mL fillable volume. The internal volumes were developed from CT-derived organ volumes [37] to provide anatomically realistic organ sizes, shapes and positions. An aluminium bar was positioned posteriorly in the phantom to simulate scatter from the spine. This set up is shown in Fig. 8.

The ratio of activity within different sections of the phantom was chosen based on clinical expectations, but the activity used was higher than would be typical clinically. Imaging time was then adjusted to simulate the clinical scenario, for example, if the expected clinical activity was 5 MBq but the phantom had a starting activity of 20 MBq, a clinical image that is typically 10 min long would be simulated by a 2.5 min equivalent acquisition. This method allowed emitted counts during the acquisition period to be accurately matched to that expected clinically, with a slight underestimation of background counts in the same scenario. Expected thyroid uptake varies substantially between patient populations, the values chosen here are reasonable estimates within a clinically expected range only.

Two scenarios were simulated;

1) **Hyperthyroid with tissue background**

Under EANM guidelines, a typical administered activity for ^99m^TcO_4_^-^ thyroid scintigraphy is 80 MBq [36]. For women in the UK population exhibiting hyperthyroidism (e.g. due to Graves’ disease) an uptake of 5% is clinically expectable [38], equating to an equivalent activity within the thyroid of 4 MBq. The background (within the approximately 5.5 L head and neck volume) to thyroid activity ratio was 1:1.9. An equivalent acquisition time of 10 min was used. Imaging distance was 65 mm and 55 mm in the anterior and lateral planes respectively. Images were acquired with the 1.20 mm, 3.07 mm and 5.00 mm pinhole collimators.

2) **Normal thyroid with hot nodule**

For an administered activity of 80 MBq [36], uptake for a normal thyroid gland is in the region of 1.5% [39], equating to an equivalent thyroid activity of 1.2 MBq. To simulate a hot nodule within a normal thyroid, an Eppendorf tube containing ~ 1 mL of ^99m^Tc was positioned behind the right thyroid lobe. The hot nodule to thyroid activity ratio was 1:6.5, giving a whole-thyroid uptake of 1.7%. An equivalent acquisition time of 10 min was used. Anterior images were acquired at 50 mm. Images were acquired with the 1.20 mm, 3.07 mm and 5.00 mm pinhole collimators. The default Seracam output is a 256 × 256 image matrix with 104 μm pixels. Acquired images were rebinned to a 128 × 128 matrix (208 μm pixels), a 64 × 64 matrix (416 μm pixels) and a 32 × 32 matrix (832 μm pixels) to explore the impact on spatial resolution and contrast.

#### Results

##### Picker thyroid phantom

**Fig. 9:**
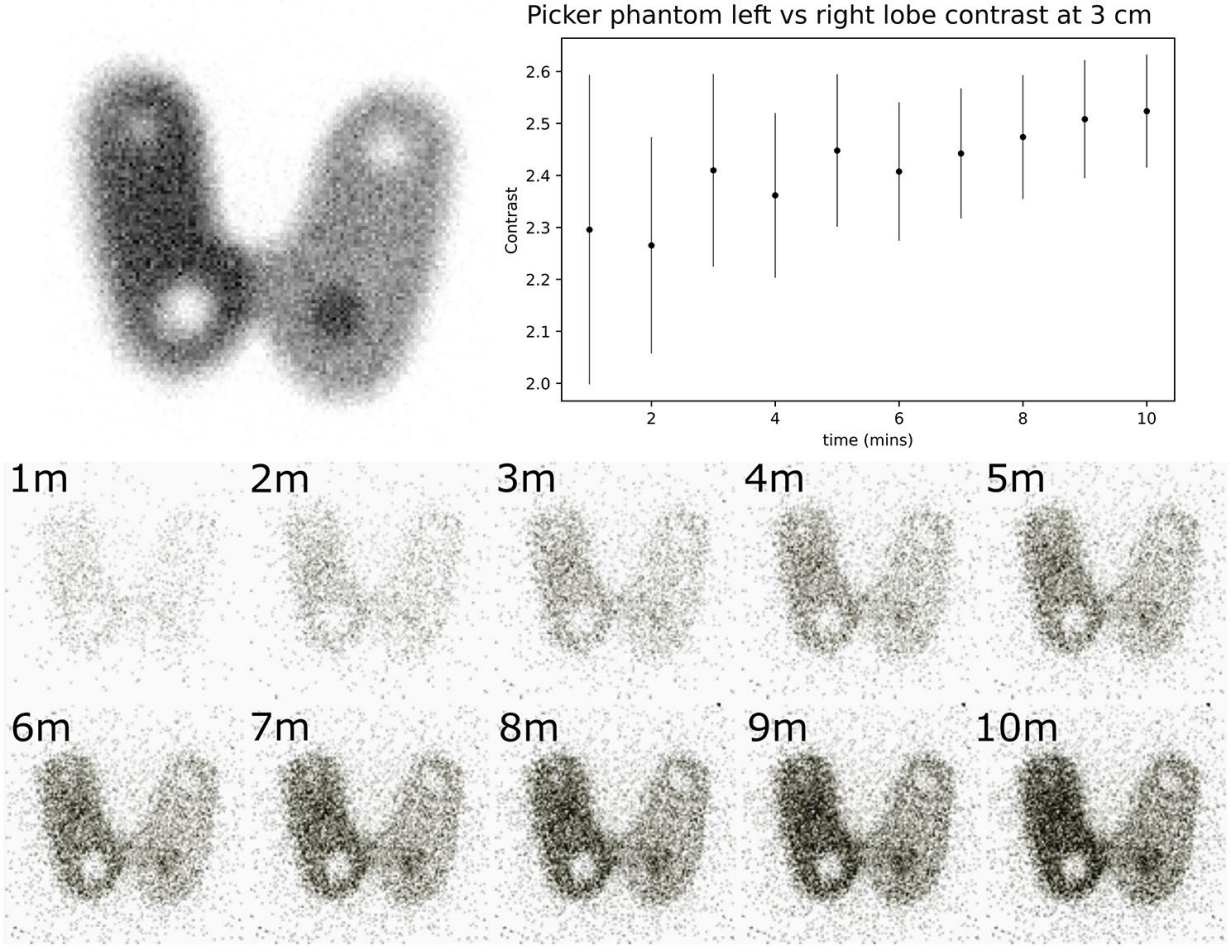
Seracam Picker phantom images. Top left: A reference Picker phantom image containing 200 kCounts, acquired on a LFOV gamma camera using a 5 mm pinhole collimator with a 128 × 128 matrix size and a zoom of 2. Reproduced from [40]. Top right: The relative contrast between the ‘hot’ left lobe and the ‘cool’ right lobe of the Seracam Picker phantom images. Based on the geometry of the Picker phantom, hot vs. cool lobe contrast is theoretically 2 although in practice the influence of scattered counts slightly elevates this value. Error bars represent counting noise. Bottom: Cumulative, 128 × 128 matrix Seracam Picker phantom images using the 1.20 mm pinhole showing the image acquired at each minute of acquisition. The phantom contained 17.5 MBq of ^99m^Tc and was imaged at 50 mm. Detected count rate was ~ 28 cps, with 17,028 counts in the 10-minute image

A time series of gamma-only Seracam images is shown in Fig. 9. The largest cold nodule is identifiable after only 2 min, with image contrast stabilising after approximately 9 min of imaging.

At an imaging distance of 50 mm (chosen to place the entire 122 mm × 112 mm phantom within the FOV) the expected spatial resolution of the 1.20 mm diameter pinhole collimator is 4.6 mm. The total number of counts acquired over 10 min was 17k, approximately an order of magnitude lower than in a typical thyroid scintigraph [36]. Despite this, these images allow for both the hot and cold 12 mm nodules and the 9 mm cold nodule to be identified. A decrease in counts in the region of the 6 mm nodule is also visible although, qualitatively, this is not clearly resolvable as a distinct feature.

##### Loughborough Head and Neck phantom

**Fig. 10:**
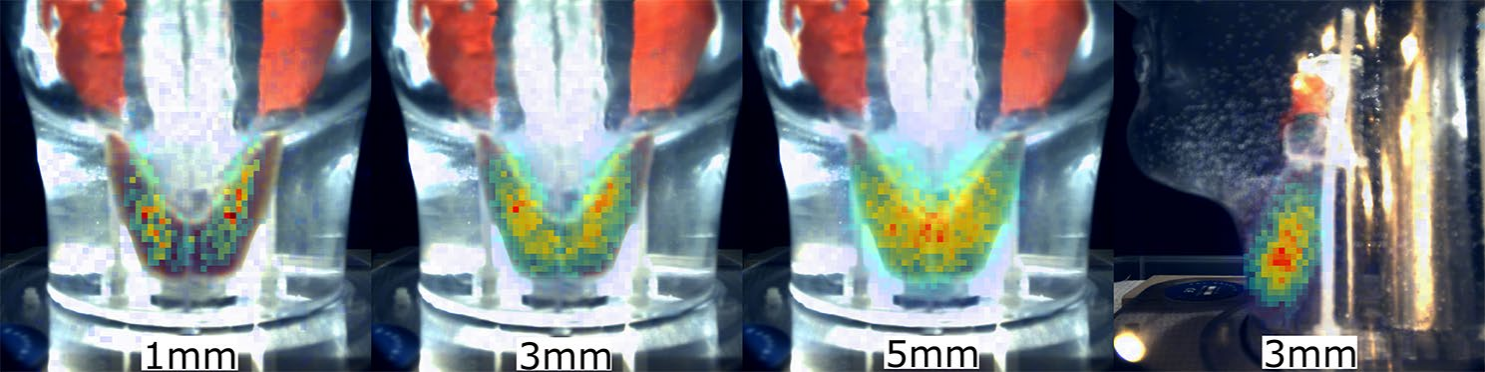
Seracam anatomical thyroid phantom images, acquired using 1.20 mm, 3.07 mm and 5.00 mm collimators. Images simulate a 10-minute acquisition of a hyperfunctioning thyroid with 5% uptake, given an 80 MBq administered activity. Total image counts are: 1.20 mm – 3450, 3.07 mm – 26,023 (anterior) and 16,895 (lateral), 5.00 mm – 68,097. Anterior images were acquired at 65 mm and lateral at 55 mm. The appearance of misregistration between gamma and optical images is due to optical refraction within the water-filled phantom

Figure 10 shows hybrid Seracam images for the hyperthyroidism simulation. The thyroid shape, location, and uniform distribution of activity is evident in all images. As expected, the smaller pinhole diameters had significantly enhanced spatial resolution, but at the expense of photon statistics for the same imaging parameters.

**Fig. 11:**
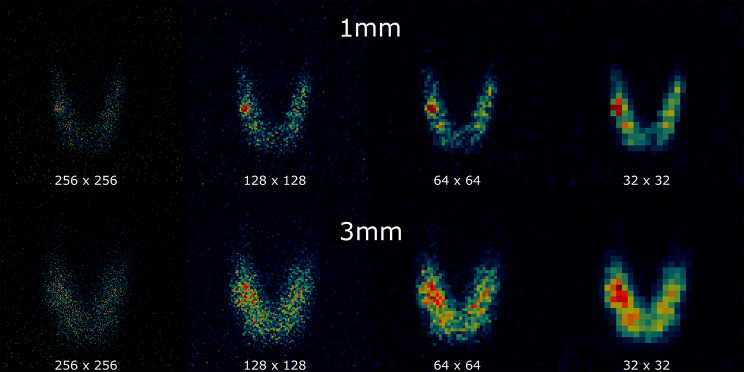
Seracam anatomical thyroid phantom images acquired using the 1.20 mm and 3.07 mm collimators, displayed using 256 × 256, 128 × 128, 64 × 64 and 32 × 32 matrix sizes. Images simulate a normal-functioning thyroid with a single hyperfunctioning nodule. Total image counts are: 1.20 mm – 3508, 3.07 mm – 6786

Figure 11 shows gamma-only Seracam images for the normal thyroid with a hot nodule configuration. The difference in imaging performance between the 1.20 mm and 3.07 mm pinhole collimator, and the effect of varying matrix size can be seen. The higher spatial resolution of the 1.20 mm collimator clearly distinguishes the hot nodule from the background thyroid activity for all matrix sizes even at relatively low counts (< 4,000). The comparatively lower spatial resolution of the 3.07 mm collimator prevents the nodule from being clearly seen on the 256 × 256 image. Coarser matrix sizes decrease the pixel noise and allow the nodule to be distinguished.

## Discussion

### Performance characteristics

The results of the performance characterisation indicate that Seracam is a suitable tool for SFOV imaging within a clinical setting.

Detected counts are linear (within 20%) up to an incident count rate of 73 kcps across the whole detector. As Seracam is an always-collimated device, this must be combined with sensitivity and spatial resolution calculations to determine the maximum activity that should be used in a particular imaging scenario. For a point source 50 mm from the camera face imaged with the 5.00 mm pinhole, count rates will be linear to at least 100 MBq of ^99m^Tc. This limit will increase for smaller pinhole sizes, greater imaging distances, or distributed sources. Clinically, activities of 4-800 MBq [1] are used depending on the imaging procedure, but the amount of activity within a localised point is extremely unlikely to reach 100 MBq. Therefore, Seracam’s CRC is suitable for routine clinical practice. Image uniformity was shown to be excellent, with little deviation from the variation expected due to photon statistics.

Seracam has four integrated pinholes, with both spatial resolution and sensitivity dependant on collimator choice. At an imaging distance of 50 mm spatial resolution ranged from 4.6 to 26 mm and sensitivity from 3.6 cps/MBq to 52.2 cps/MBq. This range of performance is comparable to clinical systems of this nature [3], particularly if the adjustment from camera distance to collimator distance is considered. For further comparison, a modern LFOV gamma imaging device (GE 870 CZT in planar mode) has a FHWM spatial resolution of 7.2–7.9 mm and sensitivity of 69–88 cps/MBq at the camera face [41].

### Clinical simulation

#### Gastric emptying

The gastric emptying simulation showed that Seracam could be used to produce a gastric emptying curve for clinically relevant activities. The imaging time used – 2 min – is higher than is currently used in traditional gamma cameras but is still short enough that it would be unlikely to add to patient discomfort. The portability of Seracam offers practical advantages. It might be possible, for example, to perform gastric emptying studies within an intensive care unit – if appropriate protocols were to be developed - and so avoid the need to transfer the patient for imaging.

At its conception, Seracam was designed with SFOV imaging in mind and for use in scenarios where its enhanced spatial resolution has clinical benefits. Gastric emptying, on the other hand, requires a larger FOV with sensitivity being the more important parameter – a challenging scenario for a system focussed on high resolution SFOV imaging. The result of this clinical simulation therefore suggests that a greater range of procedures should be explored in the future.

#### Thyroid

Both phantom scenarios have produced images of good quality and indicate that the Seracam is suitable for thyroid imaging. Qualitatively, Picker phantom image quality is similar to that achieved with a traditional LFOV camera with significantly lower counts and an imaging distance approximately 50% larger than would be used in a clinical setting.

This performance seen with the LHNP is especially promising for two key reasons. Firstly, the imaging scenarios were designed to be challenging, rather than favourable scenarios which would be expected to produce high quality images (e.g. using the average uptake in benign thyroid disease within the largest patient cohort for the hyperthyroid simulation, where uptake 4 times higher than evaluated in this study would still be clinically reasonable). Secondly, the LHNP prevented Seracam being positioned as close as would be possible in real thyroid imaging and introduces unrealistic levels of scatter due to the phantom’s 5.00 mm thick acrylic outer shell. Images of this phantom are expected to have poorer spatial resolution and counting statistics than could be achieved in the equivalent imaging of a real patient.

The hyperfunctioning thyroid simulation produced good image quality with the 3.07 mm collimator and acceptable quality with the 1.20 mm collimator. The 3.07 mm collimator successfully resolved the complete thyroid volume from the background, with sufficient counts to achieve a smooth appearance over the thyroid lobes in both the anterior and lateral planes. The quality of the 1.20 mm image was limited by its photon statistics, imaging in closer proximity to thyroid would improve this. The poorer spatial resolution of the 5.00 mm collimator resulted in a less-defined image but with far greater counts – this would not be suitable in a scenario where detailed assessment of an image is required but may have advantages when investigating uptake only (e.g. in preparation for radiotherapy).

The thyrotoxic nodule scenario replicates a low-level benign thyroid disease which would be at a preclinical level of development for most patient populations. Despite this, both the 1.20 mm and 3.07 mm collimator images achieved good quality using matrix sizes of 128 × 128 and 64 × 64. For both collimators the 256 × 256 matrix size images represented the tipping point where the counts per pixel became sufficiently low that noise substantially degraded image quality.

A key feature of Seracam images are their small pixel sizes. To contextualise the fine sampling frequency used here, a current state of the art LFOV gamma camera (GE 870 CZT) has pixel sizes across the UFOV of 1.0 mm × 0.8 mm for a 256 × 256 matrix size and 4.0 mm × 3.0 mm for a 64 × 64 matrix size [41]. The 64 × 64 matrix Seracam images would therefore be equivalent to a 512 × 512 matrix on the GE 870 CZT. The image shown here demonstrate a sampling frequency 2–4 times that currently used for thyroid scintigraphy [36].

Given the low amounts of activity used and the very fine sampling frequencies, the Seracam images obtained represent promising thyroid scintigraphy performance. Interestingly the hot nodule is easily identified with the 1.20 mm collimator using all matrix sizes. This suggests that the task of resolving the hot lesion was dominated by spatial resolution rather than sensitivity, and that this type of feature might be particularly well suited to Seracam. For patients subsequently requiring surgery, the fused gamma-optical images, showing location of nodules in relation to surface anatomy, may add further useful information for the surgeon.

### Utility assessment

Seracam has a number of novel features which are not easily quantified by NEMA-like testing or simulated clinical imaging.

As a self-contained trolley mounted system, Seracam offers patient imaging in locations inaccessible to conventional gamma cameras. This includes both rooms too small to house LFOV gamma cameras and clinical areas outside of the nuclear medicine department, such as Intensive Care Units or operating theatres. This has the potential to offer imaging to patient groups who are unable to attend the department, or where their attendance would be disruptive. The small face of the device allows it to be positioned in views which would be impossible for larger camera heads, increasing sensitivity or separating background objects. For example, during thyroid imaging patients with kyphosis are typically imaged at increased distances as they are unable to extend their neck and chests far enough to allow a LFOV gamma camera head to be positioned closely. The Seracam however could simply be placed against the patient’s neck for imaging due to the device’s small size. The flexibility offered in imaging positions, particularly in allowing a closer imaging approach, does somewhat offset the poorer sensitivity achievable with a SFOV system compared to its LFOV counterpart.

The integrated pinhole collimators are notable for how they may impact clinical practice, particularly due to the rapid speed (~ 1 s) at which they can be changed (see Sect. 2.1.2).

It is easy to imagine a future Seracam protocol using the 5.00 mm collimator for rapid, high-sensitivity area surveying or uptake measurements, interspersed with high-resolution close-up imaging of focal uptake sites with the smaller 1.20–2.22 mm collimator. This represents a different approach to imaging than is possible with LFOV gamma cameras. It appears likely that the optimal use of Seracam will require the development of specialised imaging protocols tailored for its features and capabilities.

Gamma-optical imaging (see Sect. 2.1.3) provides optical landmarking for gamma images of small organs and is particularly suitable for head and neck, paediatric imaging and intraoperative procedures. This was found to be extremely convenient during bench tests, reducing set up time and allowing for more accurate and reproducible positioning. Optical landmarking could potentially replace the use of radioactive markers in some scenarios, simplifying acquisition protocols, while also providing additional information during image reporting.

The ability to adjust colour tables and contrast on acquired images was particularly important when viewing hybrid images e.g. switching from a green-based to a red-based colour table for a green optical background. The software is intended for acquisition rather than analysis, with the ability to export images for use with standard nuclear medicine analysis software where matrix size and other parameters may be adjusted. Results of these studies suggest that the ability to change displayed matrix size during acquisition would be a valuable addition in the future.

## Conclusion

Seracam has been found to be an effective small and portable gamma camera which overcomes many of the flexibility limitations of conventional gamma cameras. Seracam demonstrated an excellent spatial resolution which significantly enhances image quality. As expected, this performance comes at the cost of reduced sensitivity when compared to LFOV devices, although the novel usability features of the Seracam and high spatial resolution capability have the promise to offset this within clinical SFOV imaging.

Given the large degree of freedom Seracam allows, with regards to both viewing angle and collimator choice, it is expected that the development of device-specific protocols will play a significant role in getting the most out of this device. As with all novel technologies which enable new approaches within established fields, it is currently difficult to predict which areas the Seracam will excel in without clinical testing. The results of this work clearly demonstrate that for SFOV imaging, Seracam offers the potential to extend the utility of nuclear medicine investigations beyond the restraints of the larger fixed camera systems.

## Data Availability

The datasets generated during this study are available from the corresponding author on reasonable request.
